# Supplementary material for: A UMLS-based spell checker for natural language processing in vaccine safety
Source: BMC Med Inform Decis Mak. 2007 Feb 12;7:3. doi: 10.1186/1472-6947-7-3 (PMC1805499; doi:10.1186/1472-6947-7-3)
Supplement: Additional file 13 — RAP application directory. Sets up directory for RAP (RDF application for PHP) source code [file 1472-6947-7-3-S13.gz › rap/doc/phpdoc/default/ATOM_RES.html]

xml version="1.0" encoding="iso-8859-1"?


Docs For Class ATOM\_RES


## Class ATOM\_RES

Description

Description |
Methods (details)

Located in /vocabulary/ATOM\_RES.php (line 21)

Method Summary

Description |
Methods (details)

void
ATOM\_CONSTRUCT
()

void
AUTHOR
()

void
CONTENT
()

void
CONTRIBUTOR
()

void
COPYRIGHT
()

void
CREATED
()

void
EMAIL
()

void
ENTRY
()

void
FEED
()

void
GENERATOR
()

void
HAS\_CHILD
()

void
HAS\_ENTRY
()

void
HAS\_LINK
()

void
HAS\_TITLE
()

void
HREF
()

void
INFO
()

void
ISSUED
()

void
LANG
()

void
LINK
()

void
MODE
()

void
MODIFIED
()

void
NAME
()

void
PERSON
()

void
REL
()

void
SUMMARY
()

void
TAGLINE
()

void
TITLE
()

void
TYPE
()

void
URL
()

void
VALUE
()

void
VERSION
()

Methods

Description |
Methods (details)

ATOM\_CONSTRUCT (line 71)

void

ATOM\_CONSTRUCT
()

AUTHOR (line 119)

void

AUTHOR
()

CONTENT (line 77)

void

CONTENT
()

CONTRIBUTOR (line 125)

void

CONTRIBUTOR
()

COPYRIGHT (line 143)

void

COPYRIGHT
()

CREATED (line 197)

void

CREATED
()

EMAIL (line 47)

void

EMAIL
()

ENTRY (line 161)

void

ENTRY
()

FEED (line 101)

void

FEED
()

GENERATOR (line 137)

void

GENERATOR
()

HAS\_CHILD (line 167)

void

HAS\_CHILD
()

HAS\_ENTRY (line 173)

void

HAS\_ENTRY
()

HAS\_LINK (line 179)

void

HAS\_LINK
()

HAS\_TITLE (line 185)

void

HAS\_TITLE
()

HREF (line 59)

void

HREF
()

INFO (line 149)

void

INFO
()

ISSUED (line 191)

void

ISSUED
()

LANG (line 113)

void

LANG
()

LINK (line 95)

void

LINK
()

MODE (line 29)

void

MODE
()

MODIFIED (line 155)

void

MODIFIED
()

NAME (line 35)

void

NAME
()

PERSON (line 83)

void

PERSON
()

REL (line 53)

void

REL
()

SUMMARY (line 203)

void

SUMMARY
()

TAGLINE (line 131)

void

TAGLINE
()

TITLE (line 65)

void

TITLE
()

TYPE (line 23)

void

TYPE
()

URL (line 41)

void

URL
()

VALUE (line 89)

void

VALUE
()

VERSION (line 107)

void

VERSION
()

Documentation generated on Fri, 17 Dec 2004 16:14:23 +0100 by phpDocumentor 1.3.0RC3
